# Experimental Infection of Pregnant Female Sheep with Zika Virus During Early Gestation

**DOI:** 10.3390/v11090795

**Published:** 2019-08-29

**Authors:** Erika R. Schwarz, Malgorzata A. Pozor, Ruiyu Pu, Kelli L. Barr, Sarah E. Beachboard, N. James MacLachlan, Dhani Prakoso, Maureen T. Long

**Affiliations:** 1 Department of Comparative, Diagnostic, and Population Medicine, College of Veterinary Medicine, University of Florida, Gainesville, FL 32611, USA; 2 Department of Large Animal Clinical Sciences, College of Veterinary Medicine, University of Florida, Gainesville, FL 32611, USA; 3 Department of Biology, Colleges of Arts and Sciences, Baylor University, Waco, TX 76798, USA; 4 Department of Pathology, Microbiology, and Immunology, School of Veterinary Medicine, University of California, Davis, CA 95616, USA

**Keywords:** Zika Virus, ZIKV, ovine, sheep, animal model, vertical transmission, pregnancy

## Abstract

Zika virus (ZIKV) is a vertically and sexually transmissible virus resulting in severe congenital malformation. The goal of this study was to develop an ovine model of ZIKV infection. Between 28–35 days gestation (DG), four pregnant animals were infected with two doses of 6 × 10^6^ PFU of ZIKV; four control animals received PBS. Animals were evaluated for 45 days (D) post-infection (PI) and necropsies were performed. Viral RNA was detected in infected ewe peripheral blood mononuclear cells (PBMC) during the first week PI; however, all fluids and tissues were negative upon culture. Anti-ZIKV IgM (1:400) and neutralizing antibodies were detected in all infected animals. Clinical disease, virus, or ZIKV antibodies were not detected in control ewes. After two weeks PI, fetal loss occurred in two infected animals, and at necropsy, three infected animals had placental petechiation and ecchymosis and one had hydramnion. Fetal morphometrics revealed smaller cranial circumference to crown-rump length ratios (*p* < 0.001) and relative brain weights (*p* = 0.038) in fetuses of infected animals compared with control fetuses. Immunophenotyping indicated an increase in B cells (*p* = 0.012) in infected sheep. Additionally, in vitro experiments using both adult and fetal cell lines demonstrated that ovine cells are highly permissive to ZIKV infection. In conclusion, ZIKV infection of pregnant sheep results in a change in fetal growth and gestational outcomes.

## 1. Introduction

Zika virus (ZIKV) is a single-stranded positive-sense RNA virus that belongs to family Flaviviridae, genus *Flavivirus*, and causes congenital malformations in children infected in utero. Termed “Congenital Zika Syndrome (CZS),” ZIKV is responsible for perinatal manifestations, including microcephaly, arthrogryposis, and ocular abnormalities [1,2]. The true long-term impact of CZS remains unknown; in Brazil, between 2015 and 2017, there were approximately 86.1 cases of CZS per 1000 cases of infected pregnant women [3]. In severe cases of CZS, the cost of care for a single child that reaches adulthood is estimated to reach $10 million USD [4]. 

To better understand the cause and effects of ZIKV vertical transmission, effective, and feasible outbred animal models are needed. Outbred models can recapitulate the disease in humans; perhaps most effective, non-human primates (NHP) have been used to develop models of both adult infection and vertical transmission of ZIKV that resembles the human disease process [5,6,7,8,9]. However, the NHP model can be inaccessible to many ZIKV researchers due to ethical and economic restraints that prevent use at many institutions. 

While mouse models have provided essential data on ZIKV pathogenesis and NHP models offer a species that most closely recapitulates human disease, sheep may provide a useful alternative. Not only are sheep outbred animals that are easy to work with, widely available to researchers, and can be used to improve statistical power (as large sample numbers are not often attainable with NHP models), but there is also evidence that sheep may be susceptible to ZIKV infection. Recent work performed in tissue culture shows that ruminant cell lines can be infected with ZIKV and are capable of sustaining infection in vitro [10,11]. Furthermore, serological studies completed in ZIKV-endemic areas indicate that ruminants, including sheep, are naturally exposed to ZIKV [12,13,14,15,16].

Sheep are recognized as an essential translational model of human pregnancy, fetal endocrine and immune ontogeny, and fetal physiology. This model is especially important as one of the few that allows for continuous fetal monitoring by in utero instrumentation [17,18,19]. Maternal and fetal telemetric or electrocardiographic leads and catheters (arterial, venous, umbilical, and amniotic) can be surgically placed to continuously monitor metabolic activity, nutrient and gas exchange, and cytokine presence, and to frequently obtain physiologic measurements and biological samples at the maternal–fetal interface [20,21,22,23]. The ability to closely monitor the maternal–fetal interface has led to the extensive use of sheep as model for human pregnancy and fetal development over the last half a century, and has contributed to an understanding of fetal stress and intrauterine growth restriction—both of which are components of maternal ZIKV infection and CZS [17,24,25,26,27,28]. The development of a model with the possibility of real-time monitoring of fetal and maternal physiology has a great potential to contribute mechanistically to the understanding of ZIKV immunopathogenesis.

Although there are some important differences between the biology and reproductive physiology of sheep and humans, certain characteristics make sheep an excellent translational model. Ovine gestation is half as long as that of humans, while still allowing for the assessment and comparison of in utero fetal growth [29]. While ovine placentas differ by classification of structure, they also share many characteristics with human placentas, especially in terms of the vascular structure and that of the villous tree [30]. The sheep conceptus does not truly invade the maternal tissue (as in humans; however, the vascular fetal allantois fuses with the chorion and creates areas of tight maternal-fetal interdigitation [31,32]. It is in these areas where the maternal tissue develops crypts into which the fetal villi grow [31,32]. Maternal to fetal transfer of nutrients and oxygen also occurs in this location, and physiologically, this is comparable between humans and sheep [17,18].

Sheep are also highly susceptible to viral teratogenic diseases, including flaviviruses, orbiviruses, bunyaviruses, pestiviruses, and others. The clinical effects of perinatal infection of sheep with the aforementioned viruses differ by the point in gestation when infection occurs. Generally, infection during the first trimester produces fetal loss, while infection during the second and third trimesters tend to produce birth defects and chronic fetal carriers [33,34,35,36,37,38,39,40,41,42]. Similarly, ZIKV infection in humans and NHP models has been shown to cause trimester-specific effects. While late-term infection most likely leads to a smaller reduction in brain growth and increased white matter gliosis, early and mid-gestation infection are more likely to result in severe microcephaly, cortical calcifications, and other significant brain pathologies [1,2,43,44,45,46,47]. Early gestation ZIKV infections have also been implicated in human and NHP fetal death and miscarriage [48,49,50]. One recent study reported a miscarriage rate of 5.8% amongst women infected with ZIKV during the first trimester of pregnancy [49]. Furthermore, evidence suggests that severe fetal effects can occur even in the absence of maternal symptoms [51].

In this study, we investigated the hypothesis that pregnant sheep are susceptible to ZIKV infection. The first goal of this feasibility study was to determine if pregnant sheep could be infected with ZIKV and whether or not fetal infection occurred. If susceptible to infection, we aimed to investigate whether or not infection during early gestation had detrimental effects on fetal growth. The second goal was to confirm the permissiveness of sheep cells to a human placental-derived strain of ZIKV.

## 2. Materials and Methods

### 2.1. Animals

All animal work was performed under the approval and guidance of the University of Florida Institutional Animal Care and Use Committee (Approval #201609345, October 14, 2016). Eight specific pathogen-free, pregnant sheep (*Ovis aries*) were purchased (Polypay breed, New England Ovis LLC, Rollinsford, NH, USA). Infected animals were housed in extended Animal Biosafety Level 2 facilities and control animals were housed separately from infected animals. Both groups of animals were housed in closed environments, free of insect vectors. Plaque reduction neutralization tests (PRNT) revealed no prior exposure to ZIKV or West Nile virus (WNV). Standard practices for feeding, husbandry, and preventive medicine were performed under the guidance of the University of Florida Animal Care Services.

### 2.2. Animal Infection and Evaluation of Clinical Disease

Four infected animals were inoculated with two doses of 6 × 10^6^ plaque-forming units (PFU)/mL of ZIKV intravenously and subcutaneously on 0 and 1 days post-infection (DPI), respectively. Back titration was performed on aliquots of ZIKV transported in the same way as the infecting inoculum to confirm the dose and stability of the virus. Four control animals received a sham inoculation of sterile PBS on each day of infection. All animals were between 36 and 42 days gestation (DG) at the time of inoculation. Mentation, appetite, rectal temperature, heart rate, respiratory rate, ruminal contractions, and capillary refill time were recorded daily and ewes were observed twice daily for fetal loss. Weekly transabdominal ultrasonography was completed to monitor the viability of the fetuses. For each visible conceptus, vesicle measurements were taken and when possible, fetal measurements and heartbeat were recorded.

Whole blood, serum, and urine were collected daily for seven DPI, then weekly, and cryopreserved at −80 °C until analysis. Peripheral blood mononuclear cells (PBMC) were isolated by lysing red blood cells (ACK Lysing Buffer, Gibco, Gaithersburg, MD, USA), washing (Hanks’ Balanced Salt Solution, Gibco), and pelleting by centrifugation at 300× *g* for 5 min at room temperature (RT), then cryopreserved at −80 °C in aliquots of 1 × 10^6^ cells in fetal bovine serum (FBS) containing 10% DMSO until use. 

### 2.3. Post-Mortem Evaluation and Tissue Collection

At 45 to 47 DPI (corresponding with 81 to 89 DG), animals were euthanized with an overdose of sodium pentobarbital and phenytoin (Beuthanasia-D, Merck Animal Health, Madison, NJ, USA) and necropsies were immediately performed. Fetal morphometric data, including body weight, brain weight, biparietal diameter, crown-rump length, cranial circumference, and femur length was recorded for all fetuses. Morphometric data of fetuses from infected ewes were compared to that of control fetuses. Tissues collected during necropsy were sectioned and snap frozen in liquid nitrogen, then stored at −80 °C until use. Sections of all collected tissues were preserved in 10% buffered formalin and embedded in paraffin. Slides were stained with hematoxylin and eosin (H&E) and histologic evaluation was performed by a veterinary pathologist.

### 2.4. Virus Quantification

A placental-derived, Asian lineage ZIKV strain R103451 (Zika Virus, R103451, NR-50355, BEI Resources, NIAID, NIH, Manassas, VA, USA) was passaged once and expanded upon acquisition in Vero-76 cells (CRL-1587, ATCC, Manassas, VA, USA) and stored in sheep serum at −80 °C. Virus was quantitated by PFU assay, in which ZIKV was mixed as eight 10-fold serial dilutions in commercial cell culture media (Modified Eagle’s Media, MEM, Gibco), inoculated into duplicate wells of a 12-well plate containing 95% confluent Vero-76 cells, and incubated at 37 °C with 5% CO_2_ for 1 h. The inoculum was removed and 1 mL of 0.05% methylcellulose overlay was added to each well. After incubation for 72 h, wells were rinsed and stained with 0.1% Coomassie blue (Thermo Fisher Scientific, Waltham, MA, USA) in 50% methanol, 43% ethanol, and 7% acetic acid for visualization. Plaques were counted and the PFU was calculated as previously described [52].

### 2.5. Virus Isolation from Animal Tissue

Maternal and fetal tissues were cultured twice as previously described [11]. Tissues were collected with a 5 mm biopsy punch and homogenized either mechanically (Qiagen TissueLyzer, Hilden, Germany or manually (Precision Tissue Grinder, Covidien, Dublin, Ireland). Large debris was removed by centrifugation and supernatant was inoculated into duplicate T-25 flasks of 80% confluent Vero-76 cells in media (MEM, Gibco) containing 1% FBS, penicillin/streptomycin, amphotericin B, and HEPES. Samples were also cultured in separate T-25 flasks of 80% confluent HEK-293 (CRL-1573, ATCC) and LLC-MK2 (CCL-7, ATCC) cells in media (Advanced DMEM, Gibco) containing 5% FBS, penicillin/streptomycin, amphotericin B, HEPES, and glutamine (GlutaMAX, Gibco). Cultures were sub-passaged at D6 PI into new flasks (P1) and both initial and P1 cultures were kept until D15 PI. Cells were monitored daily for a cytopathic effect (CPE) and, every three days, an aliquot of cells and supernatant was removed for real-time PCR (RT-PCR).

Plasma and aliquots of 1 × 10^6^ PBMC were separately cultured on 6-well plates of 95% confluent Vero-76 cells with the previously described cell culture media. For these cultures, the inoculum was left on overnight. Media was changed after 24 h, and cells were monitored daily for CPE. At D3 and D6 PI, cells and supernatant were removed for RT-PCR.

### 2.6. Real-Time PCR

RNA from serum and plasma samples were kit extracted (Zymo ZR Viral RNA Kit, Zymo Research, Irvine, CA, USA). RNA from formalin-fixed paraffin-embedded (FFPE) placentomes were kit extracted (RecoverAll Total Nucleic Acid Isolation Kit, Ambion, Austin, TX, USA) after deparaffinization (CitraSolv, Decon Laboratories, Inc., King of Prussia, PA, USA). All other samples and culture samples were extracted using a previously described guanidinium-isothyocyanate-chloroform (TRIzol Reagent, Invitrogen, Carlsbad, CA, USA) protocol [53].

RT-PCR was performed on RNA from cultures and samples of fluids and tissues (maternal: urine, plasma, serum, PBMC, placentomes, midbrain, hindbrain, uterus, mammary tissue; fetal: brain, liver, lung, kidney, spleen, amniotic fluid) using the CDC diagnostic RT-PCR protocol for ZIKV as previously described [54]. RNA from PBMC was run in triplicate; all other samples were run in duplicate. RNA from each immortalized sheep cell sample was run in triplicate. If there was disagreement between replicates, RT-PCR was repeated on the original sample. Ovine GAPDH was used as a control for the extraction of tissue RNA. Approximate quantification of ZIKV genomic copies per mL of positive sample was accomplished by comparing samples to a standard curve of synthetic ZIKV RNA template (VR-3252SD, ATCC). A known quantity (100 PFU) of pure ZIKV was used as a standardizing control on all plates.

### 2.7. Serology

#### 2.7.1. IgM Capture ELISA

An ovine IgM capture ELISA was developed based on previous methods for ZIKV IgM detection and an IgM capture ELISA protocol developed for WNV infection in the equine [55] with modification. Briefly, 96-well ELISA plates were coated with 10 µg polyclonal rabbit anti-sheep IgM (Bethyl Laboratories, Inc., Montgomery, TX, USA) in carbonate/bicarbonate buffer (pH 9.6) and incubated overnight at 4 °C. After blocking with PBS with 2% equine serum/0.5% Tween 20 for 30 min at RT, samples were diluted at 1:400 in blocking buffer (PBS with 5% milk/0.5% Tween 20) and added to duplicate wells for 1 h at 37 °C. After washing, ZIKV or negative cell culture, prepared as previously described [56], was added to each well and incubated for 1 h at 37 °C. After washing, a 1:50 dilution of anti-flavivirus antibody (D1-4G2-4-15, ATCC) in blocking buffer was added to each well and incubated for 1 h at 37 °C. Following incubation, a 1:100 dilution of sheep anti-mouse IgG-HRP (Bethyl Laboratories, Inc.) in blocking buffer was added to each well and incubated for 1 h at RT. After washing, 3,3’,5,5’tretramethylbensidine (TMB, Neogen Life Sciences, Lexington, KY, USA) was added to each well and incubated for 10 min at RT. The reaction was stopped with 2.8% sulfuric acid and plates were read using a microplate reader at 450 nm (Multiskan FC, Fisher Scientific Instruments, Co., Shanghai, China). An immune status ratio (ISR) was calculated for each specimen (ZIKV Ag OD_450_ divided by NCA OD_450_). Seropositivity was defined as an ISR ≥ 2.0.

#### 2.7.2. Plaque Reduction Neutralization Test

Serum was tested for the presence of neutralizing antibodies as previously described [57,58]. Each sample was four-fold serially diluted from 1:4 to 1:64 and mixed with an equal volume of media containing 100 PFU of ZIKV, then incubated for 1 h at 37 °C. After incubation, this mixture was inoculated into duplicate wells of a 12-well plate with 95% confluent Vero-76 cells and incubated for 1 h at 37 °C. The inoculum was removed and an overlay of media with 0.5% methylcellulose, 2.5% FBS, non-essential amino acids, penicillin/streptomycin, and amphotericin B was added to each well and incubated for three days. Plaques were stained with 0.1% Coomassie Blue in 50% methanol, 43% ethanol, and 7% acetic acid. The number of plaques in each duplicate well were counted and averaged. Antibody titers were recorded as the highest dilutions that reduced the number of plaques by at least 80%.

### 2.8. Fluorescence-Activated Cell Sorting

Fluorescence-activated cell sorting (FACS) was used to phenotype PBMC isolated from each animal, and CD4^+^ T helper, CD8^+^ T effector, γδ T cells, CD14^+^ monocytes, and B cells were quantitated. Cells were stained in duplicate using mouse anti-sheep CD45 (1.11.32, MCA2220PE, BioRad, Hercules, CA, USA), rabbit anti-sheep CD3 (SP7, ab16669, Abcam, Cambridge, MA, USA), goat anti-rabbit IgG (RRID AB_2539814, P-10994, Thermo Fisher Scientific), mouse anti-sheep CD4 (44.38, MCA2213A647, BioRad), mouse anti-sheep CD8 (38.65, MCA2216F, BioRad), mouse anti-human CD14 (TÜK4, MCA1568A700T, BioRad), mouse anti-sheep γδ T cell (WC1-N2, Washington State University Monoclonal Antibody Center, Pullman, WA, USA), goat anti-mouse IgG1 (1070-19, Southern Biotech, Birmingham, AL, USA), mouse anti-ruminant B cell (BAQ44A, S-BOV2064, Washington State University Monoclonal Antibody Center), and rat anti-mouse IgM (RMM-1, 406529, Biolegend, Waltham, MA, USA) using a previously described assay [59].

All samples were analyzed using a flow cytometer (LSR Fortessa Flow Cytometer, BD Biosciences, Franklin Lakes, NJ, USA) and commercial software (FACS Diva v8.0.1, BD Biosciences). Immune phenotypes were defined as CD4^+^ T helper cells (CD45^+^CD3^+^B^-^CD4^+^CD8^-^CD14^-^), CD8^+^ effector T cells (CD45^+^CD3^+^B^-^CD4^-^CD8^+^CD14^-^), γδ T cells (CD45^+^CD3^+^B^-^γδ^+^), monocytes (CD45^+^CD3^-^B^-^CD14^+^), and B cells (CD45^+^CD3^-^B^+^). The data for each phenotype was the average of each duplicate run, presented as the percentage of a specific cell type within the CD45^+^ gated whole cells.

### 2.9. Immortalized Sheep Cell Growth and Infection

Immortalized sheep cells, including adult kidney cells (MDOK; CRL-1633, ATCC) and fetal testicular cells (OA3.Ts; CRL-6546, ATCC) were purchased and expanded upon acquisition and passaged once in commercial cell culture media (MDOK: Eagle’s Minimum Essential Medium, ATCC; OA3.Ts: Dulbecco’s Modified Eagle’s Medium, Gibco). Upon the second passage, each respective cell line was seeded in triplicate wells of a 12-well plate and grown to 90% confluence. Additional plates were seeded with Vero-76 cells grown to 90% confluence. Each well of each cell type was infected with 50 PFU of ZIKV strain R103451 and incubated at 37 °C with 5% CO_2_ for 1 h. The inoculum was removed and 1 mL of respective culture medium was added to each well. Cells were returned to the incubator. At D1, D2, D3, D6, and D9 PI, 1 mL of cells and supernatant was removed from three wells of each infected cell line and processed for RT-PCR. Triplicate wells of non-infected cells were harvested and processed simultaneously to serve as negative controls.

### 2.10. Immunofluorescence of Immortalized Sheep Cells

ZIKV infection of MDOK, OA3.Ts, and Vero-76 cells was compared by immunofluorescence. Briefly, MDOK, OA3.Ts, and Vero-76 cells were grown in separate 6-well plates until 90% confluent. Each well of each cell type was infected with 100 PFU of ZIKV strain R103451 and incubated at 37 °C with 5% CO_2_ for 1 h. The inoculum was removed and 3 mL of culture media was added to each well. After 48 h, media was removed from each well and cells were fixed with 1 mL of 10% buffered formalin for 20 min. Formalin was removed and cells were washed three times with a permeabilization buffer (Fix and Perm Cell Fixation and Permeabilization Kit, Thermo-Fisher) and then treated with 1 mL buffer for 10 min at RT. Following treatment, the buffer was removed and 400 µL of purified mouse anti-flavivirus envelope protein antibody (D1-4G2-4-15, HB-112, ATCC) diluted in 1× PBS to 1:200 was added to each well and incubated at RT for 30 min. After incubation, the primary antibody was removed and each well was washed three times with permeabilization buffer. Then, 400 µL of Texas Red labeled rabbit anti-mouse IgG secondary antibody (ab6726, Abcam) diluted 1:200 in permeabilization buffer was added to each well. Plates were protected from light exposure and incubated at RT for 30 min. Following incubation, the secondary antibody was removed, wells were washed three times with permeabilization buffer and 400 µL of DAPI (564907, BD Pharmingen, San Jose, CA, USA) diluted 1:4000 in 1× PBS was added to each well, and plates were re-covered to prevent light exposure. Fluorescent microscopy was performed using an inverted digital microscope system (DMI 4000 B, Leica Microsystems, Buffalo Grove, IL, USA) as previously described [53], and images were visualized on commercial software (MicroPublisher 3.3 RTV, Teledyne QImaging, Surrey, British Columbia, Canada; Leica Application Suite X, Leica Microsystems). Infected wells of each cell line were compared to stained wells of uninfected cells for comparison.

### 2.11. Statistical Analysis

Statistical analysis was completed using a commercial software program (MedCalc Software bvba v18.2.1, Ostend, Belgium; http://www.medcalc.org; 2018). Significance was determined by a *p* ≤ 0.05 (α = 0.05). Morphometric data were compared between fetuses from control animals and fetuses from infected animals using a one-way analysis of variance (ANOVA) with a Levene’s test for equality of error variances and a Shapiro–Wilk test for normal distribution of residuals for each morphometric ratio. Phenotypic analysis of FACS data was analyzed for significance using repeated-measures ANOVA. 

## 3. Results

### 3.1. Clinical Disease and Fetal Development

Daily physical exams and clinical data obtained during the study revealed no overt evidence of illness or discomfort. At necropsy, minor lesions including petechiae and ecchymosis were apparent in the placentomes of three infected animals and hydramnion was present in one infected ewe. No gross lesions were noted in other organs. No microscopic abnormalities were seen in either group of maternal and fetal tissues.

**Figure 1:**
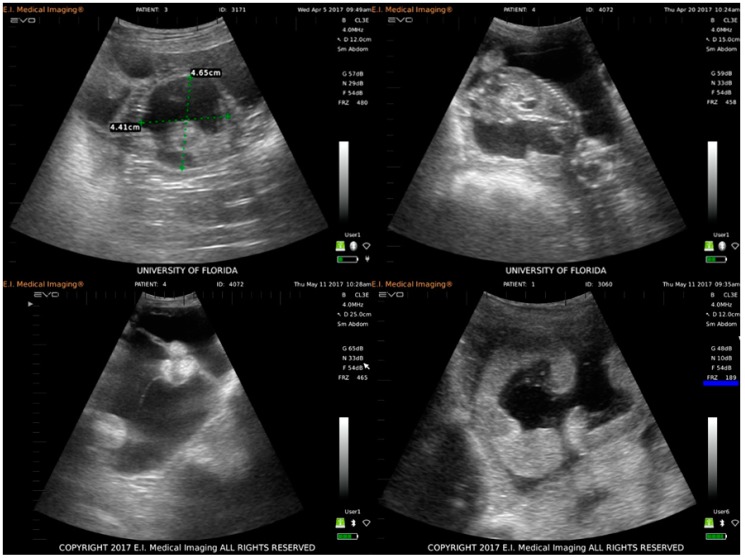
Ultrasound images of infected and control ewes taken during weekly transabdominal ultrasonography exams to monitor the viability and development of all fetuses during the infection study. Clockwise from top left: embryonic vesicle and embryo proper of a fetus from an infected animal one week post-infection (PI); lateral image of a fetus from an infected animal at four weeks PI (heart, ribcage, spine, and head are easily visualized); healthy placentomes (hyperechoic crescent-shaped object) and a normal amount of amniotic fluid in uterus of a control ewe at seven weeks PI; excess amniotic fluid accumulation (hydramnion), shrunken and misshapen placentomes (hyperechoic cluster of three round objects) consistent with hydramnion of one infected ewe at seven weeks PI.

The health and development of each fetus were monitored via weekly transabdominal ultrasonography and fetal viability was confirmed by the presence of a heartbeat. After two weeks PI, two of four infected animals experienced fetal loss. These animals initially had at least two vesicles and only one animal had more than one fetus at necropsy. No obvious fetal malformations were noted during ultrasonographic examinations (Figure 1). 

**Figure 2:**
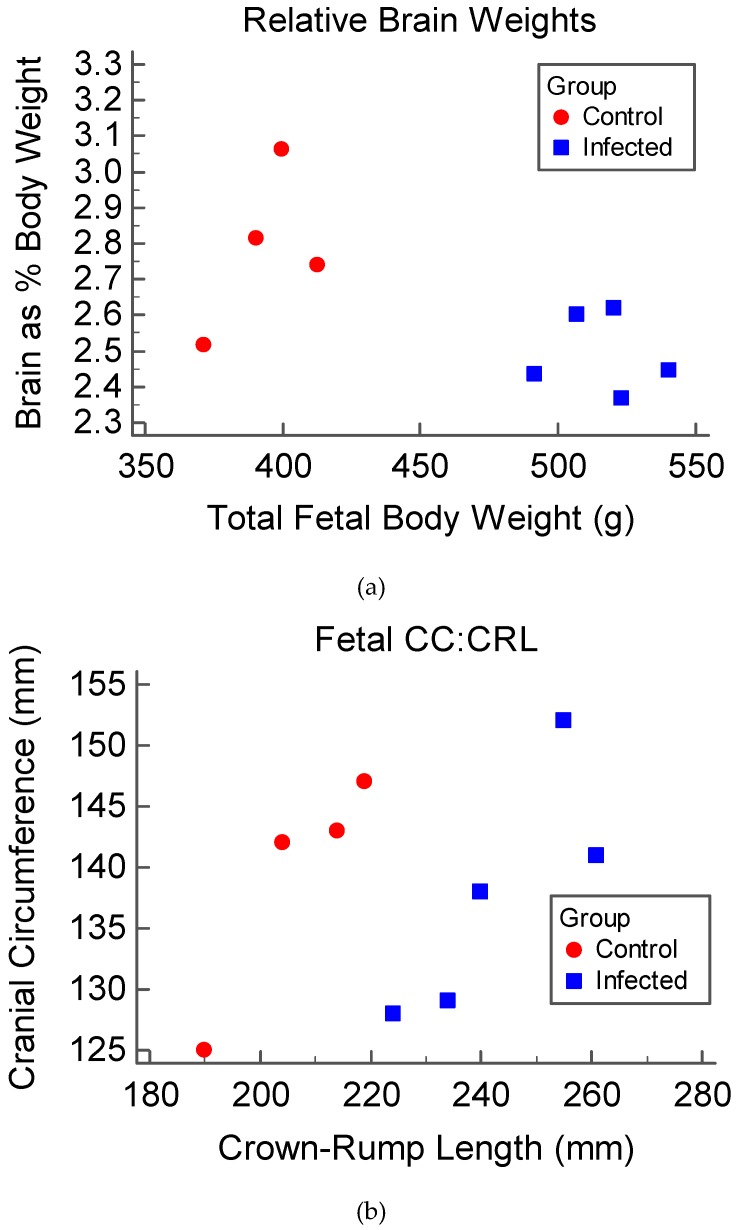
Morphometric ratios examining the brain size of fetuses at the time of necropsy (45 to 47 DPI, corresponding with a gestational age of 81 to 89 days). Morphometric ratios were calculated from fetal measurements and compared between infected (**blue**) and control groups (**red**): (**a**) The relative brain weights (brain as a percentage of body weight plotted against total body weight); (**b**) Cranial circumference plotted against crown-rump length, used to evaluate proportional head size.

At necropsy, fetal measurements were used to calculate morphometric ratios. Two morphometric ratios were used to examine brain grown compared to overall fetal growth. These ratios included relative brain weight (brain weight compared to total fetal body weight) and cranial circumference to crown-rump length (CC:CRL), both of which indicated that the growth of fetuses from infected animals did not increase proportionately during gestation. The relative brain weights (Figure 2a) for control fetuses were significantly larger than that of fetuses of infected animals (*p* = 0.038), indicating a comparatively larger proportional brain weight in control fetuses. Additionally, the CC:CRL (Figure 2b) of fetuses of infected ewes were significantly smaller when compared to that of the control fetuses (*p* < 0.001), indicating a comparatively smaller proportional head size. Cranial circumference to femur length was also calculated to investigate the presence of a femur-sparing pattern of growth restriction in fetuses of infected animals; however, this ratio was not statistically significant between groups.

### 3.2. Virus Isolation and Detection

**Figure 3:**
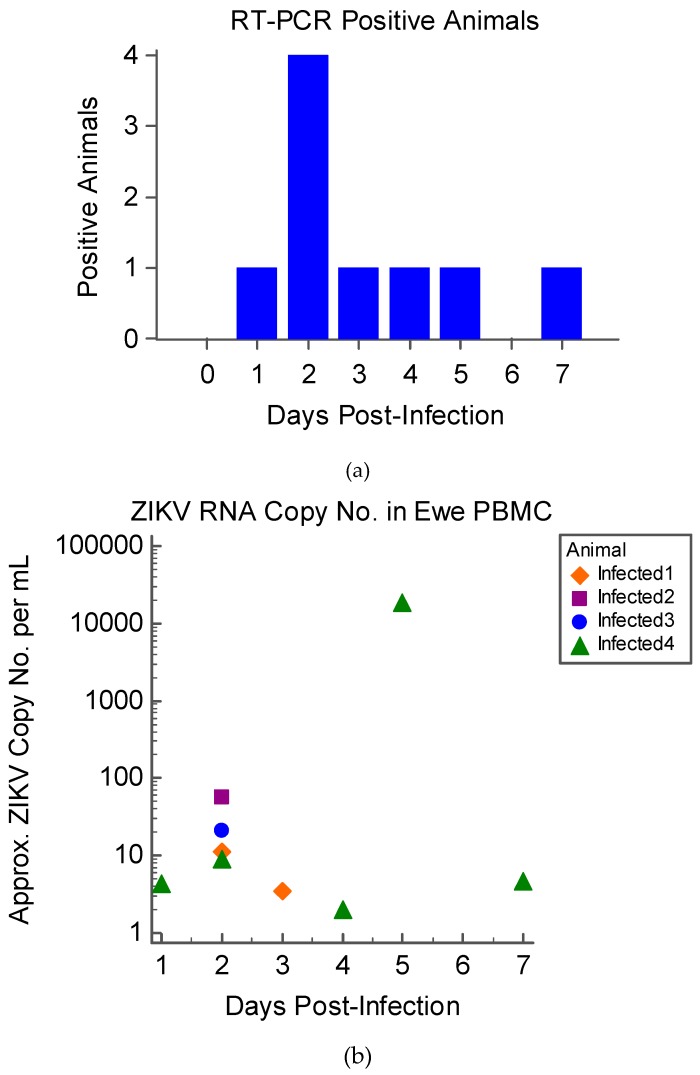
Real-time PCR results of four ewes infected with 1 × 10^6^ PFU Zika virus (ZIKV) at 36–42 days gestation: (**a**) The number of infected animals with PCR-positive peripheral blood mononuclear cells (PBMC) by DPI; (**b**) Scatterplot of the approximate copy number of genomic ZIKV RNA per mL of sample calculated for each individual infected animal by day post-infection.

The viral culture was negative for all maternal and fetal fluids and tissues. ZIKV RNA was detected by RT-PCR in PBMC of infected animals during the first week PI (Figure 3); all infected animals had ZIKV RNA positive PBMC on D2 PI (Figure 3a). None of the infected ewes were positive on D6; however, one ewe was positive on D7 PI. The ZIKV genomic copy number per 1 mL of PBMC sample from each animal ranged from 4.293 to 18,842.96 (Figure 3b). Viral RNA was not detected in any other tissue or fluid samples. All control animal samples were negative for ZIKV by culture and RT-PCR. 

### 3.3. Serology

**Figure 4:**
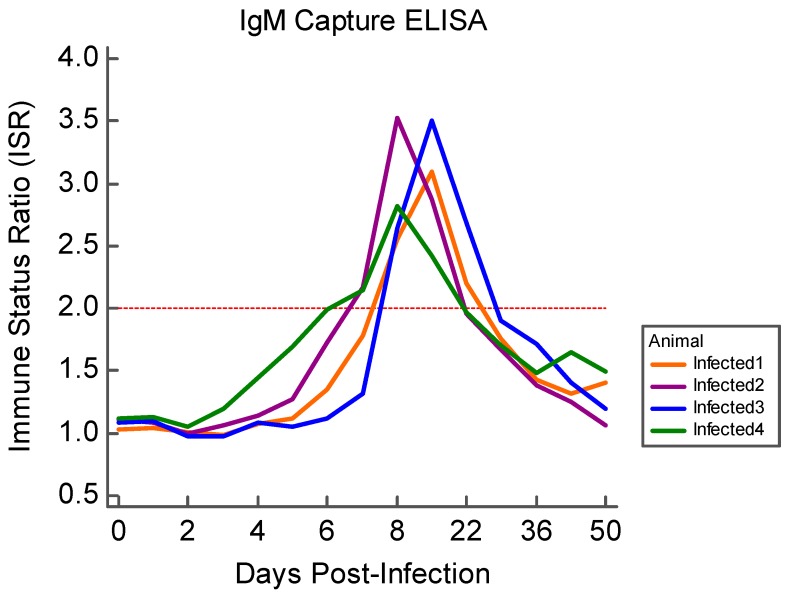
Results of the ovine specific ZIKV IgM capture ELISA for all infected animals. IgM was measured daily for the first two weeks PI, then weekly thereafter; an ISR greater than 2.0 (**red dotted line**) was considered “positive.”

Prior to the viral challenge, all animals in both groups were negative for ZIKV-specific IgM and neutralizing antibody. IgM antibody against ZIKV was first detected in infected animals by D7 PI, persisting for approximately two weeks PI (Figure 4). The mean maximum ISR value for all infected animals was 3.24 and the IgM response persisted through the first two weeks PI. All control ewes remained below the threshold for IgM detection.

**Figure 5:**
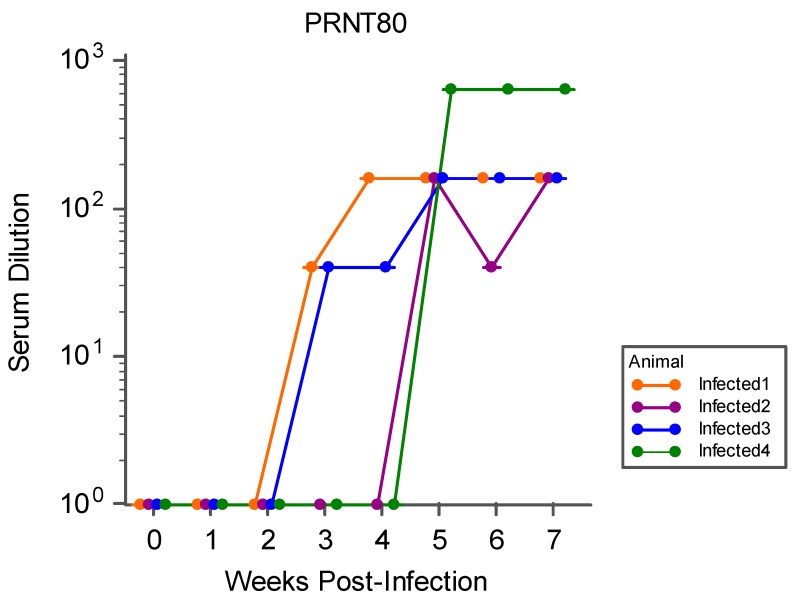
PRNT80 for all infected ewes during the study period, represented as the highest titer (serum dilution) to achieve ≥80% neutralization of viral plaque-forming units by weeks PI.

All infected animals developed neutralizing antibody by the end of the observation period, which was first detected during the second through the fourth week PI. The average geometric mean of ≥ 80% neutralization titers (PRNT80) by week five PI (Figure 5) for all infected animals was 238.49. None of the control animals developed neutralizing antibody at any point during the study.

### 3.4. Flow Cytometry

**Figure 6:**
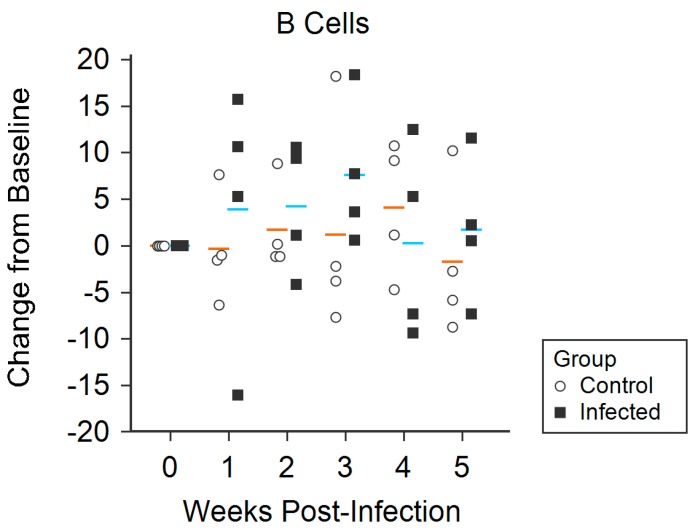
Trends of measured B cells over the course of the study period, reported as the change in percentage of cells from initial pre-infection measurement (standardized to 0 on the y-axis); horizontal lines indicate the mean change in percentage for each group, where blue represents that of infected animals and orange represents that of control animals.

Daily and weekly percentages of each PBMC phenotype were calculated for all infected and control animals. B cell percentages varied significantly between groups over the course of the study, with higher percentages being found in infected animals compared with control animals (*p* = 0.012) (Figure 6). Although there were no statistically significant trends between infection groups for CD4+ T lymphocytes, CD8+ T lymphocytes, γδ T lymphocytes, or CD14+ monocytes, the mean change in percentages of CD4+ T lymphocytes appeared to be higher in control animals when compared with infected animals (Figure S1).

### 3.5. Immortalized Sheep Cell Permissiveness to ZIKV

**Figure 7:**
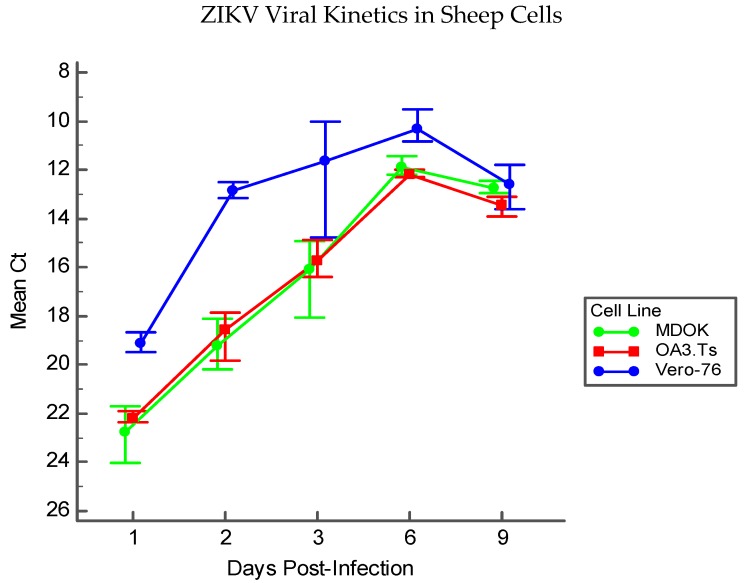
Viral kinetics of ZIKV in immortalized sheep cells measured by RT-PCR by DPI. Each point for each cell line represents the mean cycle threshold (Ct) of the three experimental replicates at each time point; error bars represent the range of Ct values in each replicate group.

Permissiveness of MDOK and OA3.Ts cells to infection with ZIKV was compared with that of Vero-76 cells by immunofluorescence and RT-PCR. Results of RT-PCR over the course of nine DPI indicate that sheep cells can sustain ZIKV infection and allow for viral replication (Figure 7). MDOK and OA3.Ts show similar permissiveness to ZIKV over all time periods, and all cell lines (including Vero-76) experience similar viral load by D9 PI.

**Figure 8:**
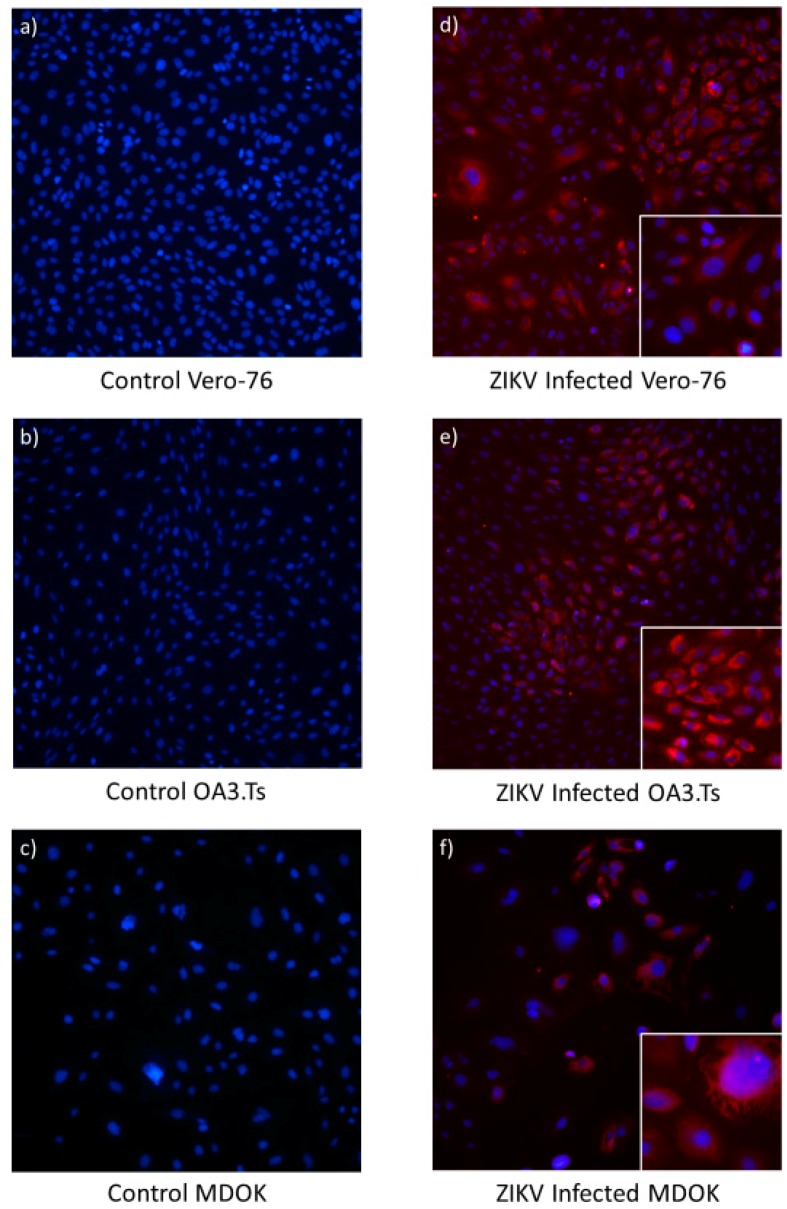
Photomicrograph comparing uninfected and ZIKV infected Vero-76 (**a** and **d**), OA3.Ts (**b** and **e**), and MDOK cells (**c** and **f**) at 48 h PI. The nuclei of both infected and uninfected cells were stained with DAPI (**blue**), while immunofluorescence (**red**) illustrates the perinuclear and cytoplasmic presence of the ZIKV E protein in infected cells (panels **d**–**f**). All large panels are shown at 10× magnification, while smaller panels in the lower right-hand corner of panels d–f illustrate individual infected cells at 20× magnification.

Immunofluorescence studies illustrated that intracellular ZIKV infection of MDOK and OA3.Ts cells are comparable to ZIKV infection of Vero-76 cells (Figure 8). By microscopic field, each infected sheep cell line showed comparable fluorescence to that of the infected Vero-76 cells, indicating similar ZIKV viral load in all cell types tested.

## 4. Discussion

The results of this study indicate that sheep are a promising model for examining the fetal effects of ZIKV. Upon infection of pregnant sheep with ZIKV, all challenged animals seroconverted, producing both IgM and neutralizing antibody to ZIKV (Figure 4 and Figure 5). Circulation of the virus, detected in PBMC by RT-PCR, preceded the development of this humoral immune response. Although the virus was not detected in whole blood or serum samples, ZIKV RNA was detected in the PBMC of infected animals over the first week PI, and all infected animals had ZIKV RNA-positive PBMC on D2 PI (Figure 3). Importantly, ZIKV infection resulted in significant fetal growth restriction evidenced by decreased relative brain weights and CC:CRL in fetuses of infected animals compared with fetuses of control animals (Figure 2). In addition, we reconfirmed that adult sheep cell lines are capable of sustaining the virus and that fetal testicular cells sustain the virus at levels at or above Vero-76 cells (Figure 7 and Figure 8). 

Although the virus was not isolated from offspring, fetal morphometrics taken at necropsy revealed that the relative brain weights and CC:CRL were significantly smaller in fetuses of infected animals when compared to those of control fetuses, suggesting that the brains of infected animals lagged in development (Figure 2). These findings are similarly seen in both human and NHP fetal ZIKV infection [2,8,27,60]. Most importantly, fetal pathology occurs in humans and multiple animal models in the absence of ZIKV vertical transmission. The findings detailed here suggest the feasibility of a large animal model that can be used to examine the indirect effects of maternal ZIKV infection on the developing fetus. 

Placental pathology, consisting of petechiation and ecchymosis, was seen in infected ewes at the time of necropsy, and at least two infected animals experienced fetal loss, which has been appreciated in both human and NHP ZIKV infection [49,61,62]. In one NHP study, 26% of infected animals experienced fetal loss following viral challenge, even though they exhibited few or no clinical signs of disease [62]. Additionally, ZIKV has been shown to cause vascular damage and subsequent insufficiency and dysfunction of the NHP placenta [63], and recent work in an immune-competent mouse model showed that placental pathology may lead to fetal effects even in the absence of clear fetal infection [64]. Given the gross placental pathology noted at the time of necropsy, ZIKV infection in the sheep may have led to vascular insufficiency of the placenta, resulting in fetal loss and growth retardation of the remaining fetuses. One infected sheep in the present study also experienced hydramnion (Figure 1), which has been implicated in human CZS [1,51,65]. Hydramnion is also a feature of Wesselbron, a flavivirus closely related to ZIKV that causes natural infection in sheep and is characterized by fetal loss and malformation [66]. Fetal loss, placental pathology, and hydramnion were also associated with the ZIKV infected sheep with the highest PBMC viral load (Figure 3). Thus, these results provide evidence of effects that have similarly been reported in other outbred animal models of ZIKV infection. Continued work with this model at high challenge doses is warranted to evaluate the consistency of ZIKV-associated pathology.

In both the sheep and macaque model, ZIKV does not appear to cause significant clinical signs of infection in the adult, which is analogous to many human infections [8,67]. Furthermore, in both humans and NHP models, the prevalence of birth defects is similar between symptomatic and asymptomatic mothers [60,62]. In NHP models, ZIKV-associated birth defects (especially neuropathology) appear to be severe and extremely common [8,68], which may not perfectly recapitulate human infection in which the prevalence of CZS is thought to be approximately 5% [69]. While ZIKV genomic RNA copy numbers in PBMC samples in the sheep study were low, this is consistent with the low-level viremia well-documented among human ZIKV patients [54,70,71,72]. Although RT-PCR does not allow for determination of replicating virus, ZIKV RNA copy number per mL detected in PBMC samples from one infected ewe fluctuated dramatically between D1 and D7 PI, implicating viral replication (Figure 3b). While viral culture from the sheep tissues was unsuccessful, this may be attributed to the nature of the tissue sampling methods. Sheep are large animals and because of the relative size of the organs, limited amounts of tissue are actually sampled. Recently, work completed in an NHP model suggested that ZIKV might cause focal infections in tissues, creating the potential for the virus to be missed during sampling [63]. However, because viral isolation from sheep tissue was unrewarding, additional studies are warranted to further adapt ZIKV to this model.

In ZIKV infected macaques, viremia is most commonly detected in the plasma and serum during the first two weeks PI and may persist for up to one month or more [9,73,74]. Interestingly, in some NHP studies, viremia could be better detected in whole blood as opposed to serum or plasma, indicating the possibility that in some cases, ZIKV is cell-associated [75]. In humans, although virus is often detectable in the plasma and serum, the primary humoral target of ZIKV is believed to be circulating CD14+ monocytes [71,76]. In our study, ZIKV was not detected in the serum or plasma; however, all of the infected sheep had RT-PCR positive PBMC during the first week PI (Figure 3a). The presence of ZIKV in ewe PBMC illustrates that viral particles were circulating in the humoral system and warrants further investigation as to the primary humoral target of ZIKV in the sheep model. Of note, none of the PBMC from the infected animals were positive on D6 PI, although one animal did have positive PBMC on D7 PI, which may indicate biphasic viremia in this animal. This animal also had the highest overall detectable viral RNA. Lack of positive samples on D6 PI could also be attributed to the time at which samples were collected; whole blood was collected only once per day, and repeated sampling may have resulted in more consistent virus detection. 

Because ZIKV was not detected in the tissue culture from infected sheep, we considered the possibility that ZIKV actually fails to replicate in ovine cells. Previous studies indicate that ruminants, including sheep, are capable of producing an immunologic response to ZIKV [12,13,14], and that ruminant cell lines are capable of sustaining ZIKV infection, suggesting the potential for viral tropism in these species [10,11]. One study has even illustrated the viral kinetics of ZIKV in a fetal sheep cell line to be extremely similar to that of *Aedes albopictus* cells (C6/36), an insect vector capable of transmitting the virus [10]. Nonetheless, we wanted to verify that ZIKV was capable of replicating in both fetal and adult sheep cell lines under similar culture conditions as the in vivo experiments. The permissiveness of two different immortalized sheep cell lines (MDOK and OA3.Ts) to ZIKV infection were tested and both the adult MDOK and the fetal OA3.Ts cells were capable of sustaining infection over many days (Figure 7). Viral load was similar in both ovine cell lines compared to Vero-76 cells, and presence of ZIKV was easily visualized in all cell lines tested with immunofluorescence targeting the envelope protein after 48 h PI (Figure 8). Taken together, these experiments suggest that sheep cells are in fact permissive to infection and sheep may be a susceptible ZIKV host species. 

PBMC phenotyping was suggestive of recruitment of specific cell types. Relative mean changes in the percentage of B cells were significantly increased (*p* = 0.012) amongst infected animals during the observation period (Figure 6). The relative increase in B cells of infected animals from weeks one to three PI is contrasted with that of the control animals, which did not fluctuate during the observational period. Although changes in other PBMC phenotype percentages (CD4+ T lymphocytes, CD8+ T lymphocytes, γδ T lymphocytes, CD14+ monocytes) were not statistically significant (Figure S1), a small overall reduction in CD4+ T lymphocytes was detected in the infected animals (Figure S1a). Ruminants, including sheep, frequently experience a reduction in peripheral lymphocytes in response to stress and viral infection, while changes in peripheral monocytes are less evident [77,78,79]. It is also worth noting that because all of the animals in this study were outbred, PBMC phenotypes were expected to vary widely, which can be overcome with studies using larger numbers of animals. 

Given that sheep are a well-established model for human pregnancy and fetal stress, easy to work with, and offer the opportunity to monitor the maternal–fetal interface with in utero instrumentation, the continued development of an ovine infection model would greatly benefit researchers by offering a unique system in which to study the host–pathogen interactions and perinatal effects of ZIKV in the outbred host. The results of this feasibility study suggest that sheep are susceptible to ZIKV and that fetal effects are seen post-infection; thus, with continued development, sheep may offer a promising animal model for studying ZIKV vertical transmission and subsequent perinatal effects. Additional studies are currently underway to modify and improve the utility of this ovine infection model.

## Supplementary Materials

The following are available online at https://www.mdpi.com/1999-4915/11/9/795/s1, Figure S1: PBMC phenotypes measured by FACS over the course of the study period, reported as the change in percentage of cells from initial pre-infection measurement (standardized to 0 on the y-axis); horizontal lines indicate the mean change in percentage for each group, where blue represents infected animals and orange represents control animals. Specific phenotypes measured included: (a) CD4+ T lymphocytes; (b) CD8+ T lymphocytes; (c) CD14+ monocytes; and (d) γδ T lymphocytes.
