# Supplementary material for: Experimental Infection of Pregnant Female Sheep with Zika Virus During Early Gestation
Source: Viruses. 2019 Aug 29;11(9):795. doi: 10.3390/v11090795 (PMC6784126; doi:10.3390/v11090795)
Supplement: Supplementary file 1 [file viruses-11-00795-s001.pdf]

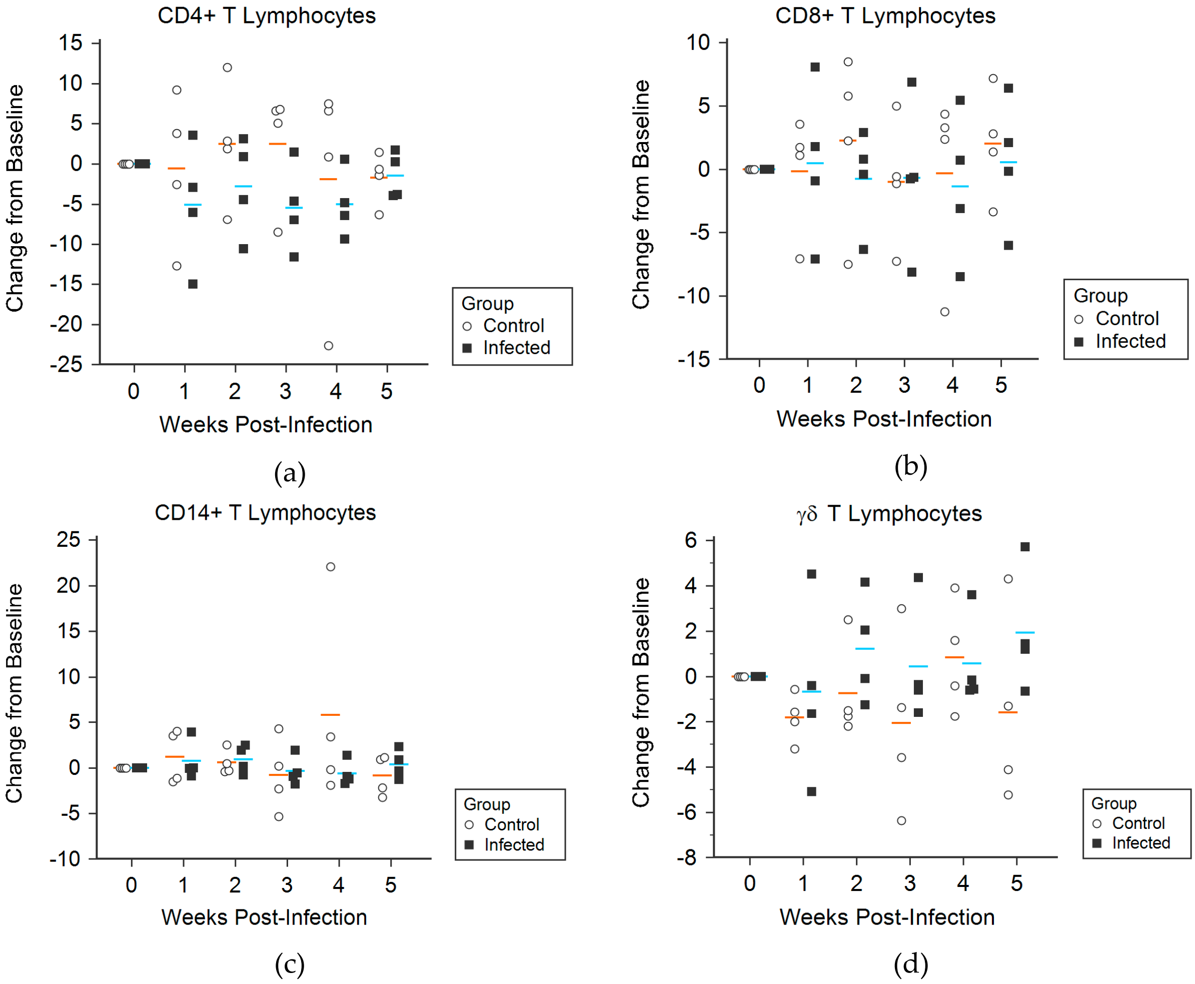


**Figure S1.** PBMC phenotypes measured by FACS over the course of the study period, reported as the change in percentage of cells from initial pre-infection measurement (standardized to 0 on the y-axis); horizontal lines indicate the mean change in percentage for each group, where blue represents infected animals and orange represents control animals. Specific phenotypes measured included: a) CD4+ T lymphocytes; b) CD8+ T lymphocytes; c) CD14+ monocytes; and d) γδ T lymphocytes.
